# MALDI-TOF mass spectrometry from nucleic acid: development and evaluation of a novel platform for identification of mycobacteria and detection of genetic markers of resistance

**DOI:** 10.1128/spectrum.01638-24

**Published:** 2024-08-27

**Authors:** Emily K. DeCurtis, Iara Machado, Sharon K. Kuss-Duerkop, Yongbao Wang, Reeti Khare

**Affiliations:** 1 Advanced Diagnostics Laboratory, National Jewish Health, Denver, Colorado, USA; 2 Department of Medicine, National Jewish Health, Denver, Colorado, USA; University of Maryland School of Medicine, Baltimore, Maryland, USA

**Keywords:** PCR, MassArray, MALDI-TOF MS, mycobacteria, *rrs*, *rrl*, *erm *(41), NTM

## Abstract

Complete identification methods are critical for evaluating nontuberculous mycobacteria (NTM). Here, we describe a novel diagnostic method for identification of eight NTM, *Mycobacterium tuberculosis* complex, and three drug resistance markers using PCR/matrix-assisted, laser-desorption ionization time-of-flight mass spectrometry (MALDI-TOF MS) from cultured organisms. With this technology, a multiplex end-point PCR is performed for targets of interest. Detection probes that are extended in the presence of a target are added. The extended probes have greater molecular weight and can be detected by MALDI-TOF MS. An AFB Primary Panel was designed to differentiate *Mycobacterium avium*; *Mycobacterium intracellulare* subsp. *chimaera*; *Mycobacterium avium* complex (other); *Mycobacterium abscessus* subsp. *abscessus*, *bolletii*, and *massiliense*; *Mycobacterium kansasii*, and *M. tuberculosis* complex. This design should cover 90% (3,483/3,691) of mycobacteria seen onsite. A development set of unblinded isolates (*n* = 217) was used to develop PCR primers, detection probes, and probe barcodes. It demonstrated 99.1% (215/217) agreement with reference methods. An evaluation set using blinded isolates (*n* = 320) showed an overall sensitivity of 94.3% (range by target: 90.0–100%). Overall specificity from negative media, non-target mycobacteria, and bacteria was 99.1% (108/109; range by target: 94.4–100%). Three drug resistance markers *erm* (41), *rrl,* and *rrs* demonstrated 100%, 91%, and 100% sensitivity, respectively, and >99% specificity. Limit of detection per target ranged from 2.2 × 10^3^ to 9.9 × 10^6^ CFU/mL. The AFB Primary Panel allows for mycobacterial speciation, subspeciation, and resistance mutation detection, which is essential for diagnosis, appropriate therapy, identifying outbreaks, and managing treatment-refractory disease. It can perform with high-throughput and high specificity and sensitivity from isolates.

**IMPORTANCE:**

Even closely related mycobacteria can have unique treatment patterns, but differentiating these organisms is a challenge. Here, we tested an innovative platform that combines two commonly used technologies and creates something new: matrix-assisted, laser-desorption ionization time-of flight mass spectrometry was performed on PCR amplicons instead of on proteins. This created a robust system with the advantages of PCR (high discriminatory power, high throughput, detection of resistance) with the advantages of mass spectrometry (more targets, lower operational cost) in order to identify closely related mycobacterial organisms.

## INTRODUCTION

Nontuberculous mycobacteria (NTM) are environmental organisms that can cause opportunistic pulmonary, tissue, and disseminated infections. These infections have a higher prevalence in immunocompromised populations and those with chronic obstructive pulmonary disease, cystic fibrosis, or other respiratory diseases (1, 2). Notably, the prevalence of NTM infections is increasing (3–6).

Currently, over 190 species of NTM have been identified, but only a few organisms represent the majority of human infections in the United States, such as species in the *Mycobacterium avium* complex and *Mycobacterium abscessus* subspecies (5–7). Here, we evaluated the performance of a laboratory-developed assay for accurate identification of nine important mycobacteria and three genes associated with drug resistance from pure cultured isolates (8–10). Testing was performed on a novel platform called polymerase chain reaction (PCR)/matrix-assisted, laser-desorption ionization time-of-flight mass spectrometry (MALDI-TOF MS).

In this methodology, a multiplex end-point PCR is performed first for amplification of target genes. This is followed by a second end-point amplification reaction during which single-stranded detection probes are added. If the target-of-interest amplicons are present, the detection probes will bind and are extended by a single base. This results in detection probes of an expected molecular weight. We then used MALDI-TOF MS to detect the presence of these extended detection probes.

Compared to MALDI-TOF MS alone, the addition of PCR prior to MALDI-TOF MS allows for detection of drug resistance as well as a greater ability to speciate and subspeciate mycobacteria. Complete identification is essential for several reasons. Firstly, diagnosis of NTM in sputum requires two specimens that are positive for precisely the same organisms (5). Secondly, even closely related mycobacteria have significantly different susceptibilities. For instance, *M. abscessus* subsp. *abscessus* commonly harbors a functional *erm* (41), the gene that confers inducible clarithromycin resistance (11, 12), while the *erm* (41) gene is known to be truncated in *M. abscessus* subsp. *massiliense*, and this organism is not subject to inducible clarithromycin resistance (11, 13, 14). Finally, accurate identification of these mycobacteria assists in evaluating the etiology of infections (e.g., outbreaks or re-infections versus treatment-refractory infections) (15, 16).

## MATERIALS AND METHODS

### Organism prevalence

The number of mycobacteria and partially acid-fast organisms reported from National Jewish Health (NJH) patients tested in the NJH Mycobacteriology Laboratory from 01 January 2020 to 01 January 2022, inclusive, was retrieved from the laboratory information system (SoftMicLab, Soft Computer Consultants Inc, Clearwater, FL, USA).

### Study design for accuracy and cross-reactivity testing

Pure isolates were tested by the AFB Primary Panel (Table 1). Isolates were chosen randomly or purchased from the American Type Culture Collection (ATCC), e.g., *Escherichia coli* ATCC 25922, ATCC 35218, and *Staphylococcus aureus* ATCC 25923. The organisms identified by the AFB Primary Panel are *M. abscessus* subsp. *abscessus, M. abscessus* subsp. *bolletii*, *M. abscessus* subsp. *massiliense*, *M. avium sensu stricto*, *M. intracellulare* subsp. *chimaera*, *M. avium* complex (other), *M. chelonae*, *M. kansasii,* and *M. tuberculosis* complex (MTBC). If the panel cannot differentiate an organism, then the result of “No Identification” is given. The drug resistance targets included the detection of a functional (C28 mutation) and nonfunctional (T28 mutation) *erm* (41) for inducible macrolide resistance, mutations in the 2058 and 2059 positions of *rrl*, and mutations in the 1408 and 1409 positions of *rrs* for resistance to macrolides and aminoglycosides, respectively. All other organisms tested for cross-reactivity are listed in Table S1.

**TABLE 1:** Study design for targets on the AFB Primary Panel evaluated for accuracy and cross-reactivity^*b*^

Target defined on panel	Genes targeted	Reference standard identification method	Relevant drug markers
*M. abscessus* subsp. *abscessus*	Thymidylate kinase, β-galactosidase, *ku*	LPA, *hsp65* sequencing	*erm* (41), *rrl*, *rrs*
*M. abscessus* subsp. *bolletii*	Thymidylate kinase, β-galactosidase, *ku*	LPA, *hsp65* sequencing	*erm* (41), *rrl*, *rrs*
*M. abscessus* subsp. *massiliense*	Thymidylate kinase, β-galactosidase, *ku*	LPA	*rrl*, *rrs*
*M. avium sensu stricto*	*rpoB*, ITS, *ku*	LPA	*rrl*, *rrs*
*M. intracellulare* subsp. *chimaera*	*rpoB*, ITS, *ku*	LPA	*rrl*, *rrs*
*M. avium* complex, other^*a*^	*rpoB*, ITS, *ku*	LPA	*rrl*, *rrs*
*M. chelonae*	*rpoB*, *ku*	LPA	*rrl*, *rrs*
*M. kansasii*	*rpoB*, ITS, *ku*	*rpoB* and 16S sequencing	N/A
*M. tuberculosis* complex	*mce3B*, *IS6110*, *ku*	LPA, *rpoB* and 16S sequencing	N/A
*erm* (41)	C28T mutation	LPA/AST	N/A
*rrl*	Mutations at 2058 and 2059	LPA/AST	N/A
*rrs*	Mutations at 1408 and 1409	LPA/AST	N/A

^*a*^ *“M. avium* complex, other” is defined as any organism not identifiable beyond the complex level by the AFB Primary Panel.

^*b*^ AST: antimicrobial susceptibility testing, ITS: internal transcribed spacer region, IS: insertion sequence, LPA: line probe assay, N/A: not applicable.

A range of organisms including mycobacteria (representing 32 species or subspecies), partially acid-fast organisms (four species), and bacteria (three species) was used. Of these, a development set (*n* = 217) of isolates was tested in an unblinded manner to create the MassArray Reference File and the Pattern Definition File (see Result Definition section below). The remainder (*n* = 320) was used as an evaluation set and tested in a blinded manner. Discrepants between the AFB Primary Panel and routine (reference) identification methods were repeated or retested by another method.

### Methods of identification

GenoType MTBC VER 1.X (Hain Lifescience, Bruker, Nehren, Germany) was used to identify the species of MTBC. The GenoType NTM-DR version 1.0 (Hain Lifescience, Bruker, Nehren, Germany) line probe assay was used to routinely identify the following species, according to manufacturer's instructions: *M. avium*, *M. intracellulare* subsp. *chimaera* (17, 18), *M. abscessus* subsp. *abscessus*, *M. abscessus* subsp. *bolletii*, *M. abscessus* subsp. *massiliense*, and *M. chelonae*; mutations in *rrl* and *rrs*; and the presence of functional *erm* (41) resistance markers. *M. intracellulare* subsp. *intracellulare* and other mycobacteria were routinely identified using laboratory-developed Sanger sequencing assays described elsewhere (19) due to known cross-reactivity of this target with other *M. avium* complex members (20, 21). In brief, isolates from solid or liquid culture were resuspended in Prepman Ultra Sample Preparation Reagent (Life Technologies), heat-inactivated, and centrifuged to separate out cellular debris. PCR amplification was performed on the supernatant with primers specific to a 761 bp region within *rpoB*, a 500 bp region within the 16s rDNA gene, and a 413 bp region within *hsp*65. Amplicons were dephosphorylated and sequenced in both the forward and reverse directions with the ABI 3130xl Genetic Analyzer (Applied Biosystems, Bedford, MA, USA). The *rpoB* and 16S sequencing results for slowly or rapidly growing NTM were analyzed as described previously (19). The analysis of the *hsp*65 gene sequencing data was performed by aligning the sequencing results with the reference mycobacteria sequence in the Geneious Prime software version 2022.1.1 (Geneious, Auckland, New Zealand) and/or by performing a NCBI blast of the *hsp*65 result sequence. The identification result determination from GenBank following the previously described quality requirements as well as >99% sequence similarity.

### Nucleic acid extraction for PCR/MALDI-TOF MS

Culture, heat inactivation, and DNA extraction was performed in a biosafety level (BSL3) laboratory. Downstream identification and susceptibility testing for NTM and MTBC was performed in BSL2 and BSL3 conditions, respectively. Isolates were streaked for isolation using Middlebrook 7H11 or 7H11S, chocolate, or Lowenstein-Jensen (Remel, Lenexa, KS, USA) solid media. Single, isolated colonies were either extracted directly from solid media or further cultured in 7H9 (Remel) or MGIT broth (BD Biosciences, Franklin Lakes, NJ, USA). Cultures with slowly growing mycobacteria and *M. tuberculosis* complex were incubated at 37°C, *M. xenopi* was incubated at 42°C, and rapidly growing mycobacteria were incubated at 32°C. Liquid cultures were grown to a 0.5 McFarland standard, and then 1 mL was centrifuged at 12,000 × g for 15 minutes. The supernatant was discarded, and the pellet was resuspended in 200 µL of Prepman Ultra. From solid media, single colonies (2–3 mm) were suspended directly in 200 µL of Prepman Ultra.

Prepman Ultra suspensions from both sample types were vortexed for 15 seconds or until homogenous, heat-killed for 30 minutes in a 90–100°C water bath, and centrifuged again at 12,000 × g for 15 minutes. A 200 µL aliquot of the supernatant was stored either refrigerated (2–8°C) or frozen (−25°C). Prior to testing, extracts were diluted 1:10 into nuclease free water.

### PCR/MALDI-TOF MS

**Fig 1:**
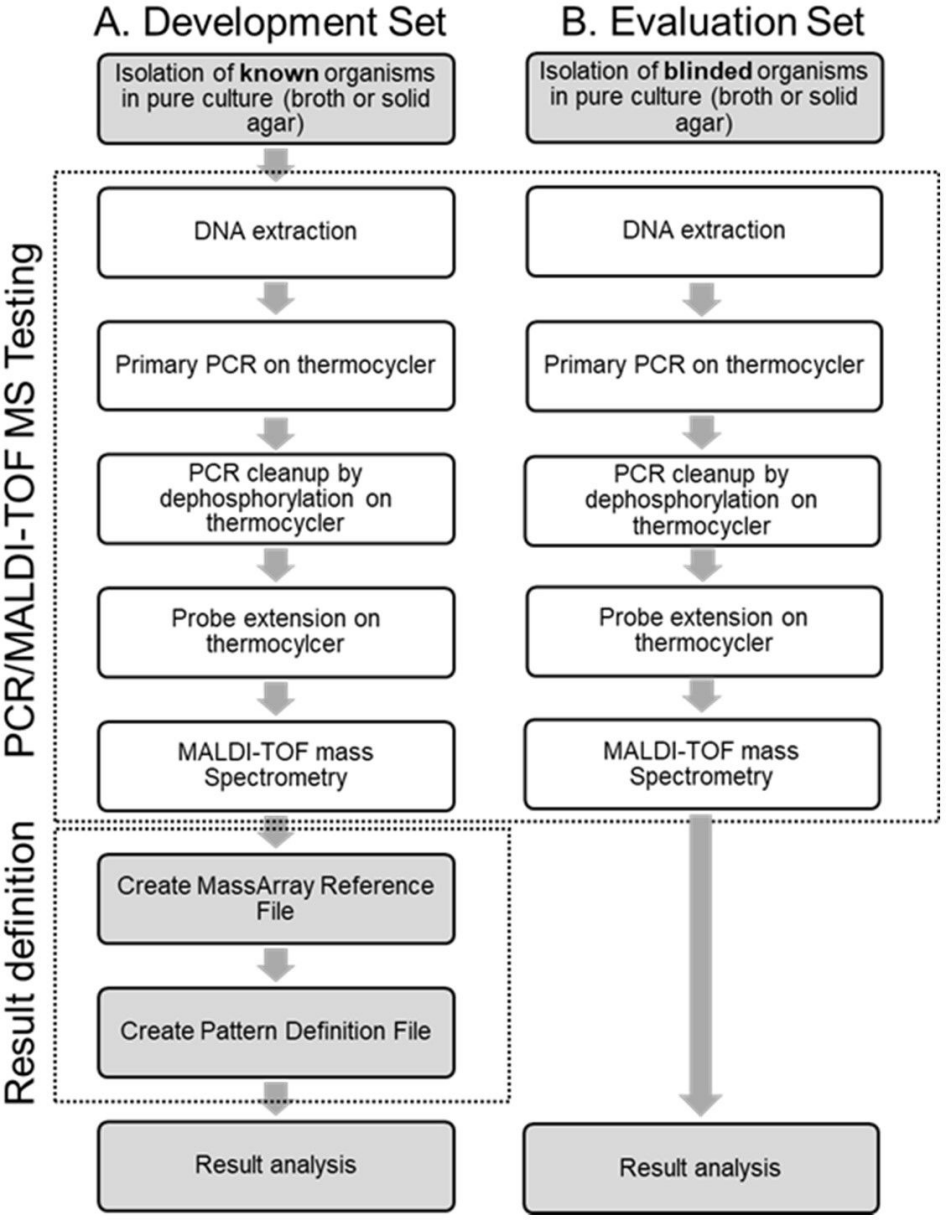
Workflow for PCR/MALDI-TOF mass spectrometry (a) during design and evaluation with known isolates and (b) during testing of the panel with unknown isolates.

The overall testing methodology is shown in Fig. 1. First, a multiplex PCR reaction was performed to amplify 24 target regions in 10 different genes (Table 1). Extracted DNA (2.5 µL) was combined with 1.99 µL of mastermix (PCR Reagent Kit, Agena Bioscience, San Diego, CA, USA), 1.66 µL of a proprietary primer pool (IDT Technologies, Coralville, IA, USA), 1.27 µL nuclease free water (Invitrogen, Waltham, MA, USA), and 0.08 µL of 3.125 mg/mL amaranth dye (Sigma-Aldrich, St. Louis, MO, USA). Cycling conditions were as follows: 2 minutes at 95°C for 1 cycle, 5 cycles of 95°C for 30 seconds, 25 cycles of 60°C for 30 seconds and 72°C for 1 minute, 25 cycles of 30 seconds at 95°C and 30 seconds at 65°C, 5 cycles of 1 minute at 72°C, 1 cycle of 5 minutes at 72°C, and a holding temperature of 4°C.

A PCR clean-up to dephosphorylate unincorporated nucleotides was performed using 5 µL of PCR amplicons, 0.47 µL of shrimp alkaline phosphatase master mix (IPLEX Reagent kit, Agena Bioscience), 1.405 µL of nuclease free water, and 0.125 µL of 20× bromphenol blue (3.13 mg/mL; Sigma-Aldrich). Incubation conditions were as follows: 1 cycle of 37°C for 10 minutes and 1 cycle of 85°C for 5 minutes with a final holding temperature of 4°C.

Following this, a second multiplex PCR reaction was performed to extend detection probes by a single ddNTP (Agena Bioscience). The cleaned PCR product (7 µL) was added to 0.44 µL of IPLEX Pro Reagent Set master mix Extension Reaction reagents (Agena Bioscience) combined with 0.57 µL of nuclease free water, 0.94 µL of a pool of proprietary detection probes (IDT Technologies), and 0.05 µL of 60× xylene cyanol (3.2 mg/mL; Sigma-Aldrich). Cycling conditions were as follows: 1 cycle of 30 seconds at 94°C, 40 cycles of 5 seconds at 94°C, 15 seconds at 52°C, and 5 seconds at 80°C. A final cycle of 72°C for 3 minutes was performed, with a holding temperature of 4°C.

The products from the extension reaction were brought to a 50 µL volume using nuclease free water (Invitrogen) and were loaded onto MALDI-TOF MS (MassARRAY, Agena bioscience). To clean the samples, the automated Chip Prep Module (Agena Bioscience, SpectroAcquire V4.3.145) dispensed resin into the samples. This module also spotted the cleaned product onto a silicon chip that was preloaded with a crystal matrix.

### Result definition

In the design phase, a MassArray Reference File was created to define the mass/charge ratio of each extended and unextended probe (Fig. 1). It also defines the expected nucleotide call for each extended probe. Note that the baseline thresholds were calculated by evaluating a no template control (nuclease free water) as a sample and analyzing the baseline intensity for each targets extended probe's nucleotide peak result.

The nucleotide that is “called” for each probe represents the single ddNTP that was added to the extension probe. The individual nucleotide calls for all the probes are concatenated to form a unique pattern, or barcode, for each species/subspecies and drug resistance target. The barcodes from known organisms were used to create a database for each target on the AFB Primary Panel called the Pattern Definition File (Fig. 1). This Pattern Definition File is used by the MassArray Typer Analyzer software v4.10.1.5 (Agena Bioscience) for final identification. If a sample produces a “not defined barcode” then the sample will result in a “No Identification” result and reflexed for further testing.

### Result analysis

The ratio of extended to unextended mass/charge is calculated either manually (by comparing peak intensities) or automatically by the Reporter Module in the MassArray Typer Analyzer. If the extended to unextended ratio is above the defined threshold for a specific target in the Reporter Module, the result is recorded as positive for that target, and the software will report the nucleotide call. If it is below the defined threshold, the result is recorded as negative for that target.

### Limit of detection

The limit of detection (LOD) for each target on the AFB Primary Panel was determined. A 0.5 McFarland was made for each target on the panel, except for *M. abscessus* subsp. *abscessus* where two strains were tested. The isolate suspensions were prepared in demineralized water. Six additional 10-fold dilutions were created for each suspension. All concentrations were plated and colonies were counted to determine the colony forming units per milliliter (CFU/mL) for each culture. To determine the approximate LOD range, we extracted each concentration three separate times and tested it in duplicate PCR reactions. To determine the final LOD for each target on the panel, we tested 20 replicates, and the limit of detection was determined to be the concentration (CFU/mL) at which ≥95% of replicates were detected. Where more than one isolate was tested for a specific target, the final LOD was determined by averaging the LODs determined for each isolate.

### Statistical analysis

Confidence intervals were calculated using Graphpad Quick Calcs interval of a portion calculation (modified Wald method) for identification and drug resistance sensitivity and specificity results (https://www.graphpad.com/quickcalcs/confInterval1/).

## RESULTS

**Fig 2:**
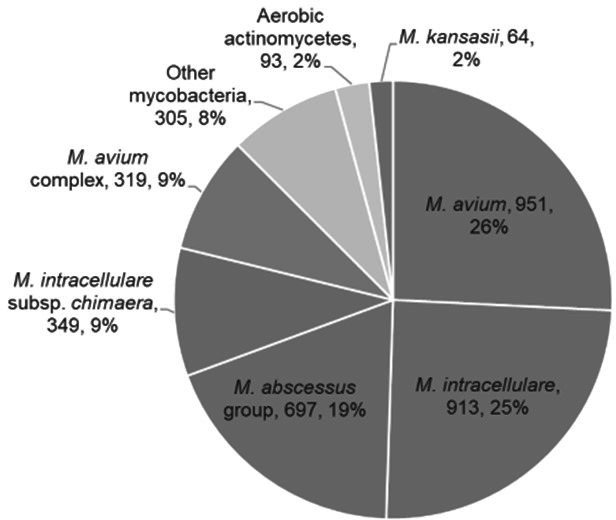
Species Identified at the National Jewish Health mycobacteriology laboratory (*n* = 3,691) across 2 years. The species shown in dark gray include organisms represented on the AFB Primary Panel.

From 01 January 2020 to 01 January 2022, we reported 3,691 organisms by the laboratory. Of these, *M. avium* complex accounted for 69% (2,532/3,691) of all organisms identified, with the most commonly identified organism being *M. avium sensu stricto* (26%; 951/3,691). Of all mycobacterial organisms identified within the 2-year period, 90% (3,483/3,691) would be identifiable using the design of the AFB Primary Panel (Fig. 2).

A total of 537 isolates were tested by the AFB Primary Panel. Of these, 217 isolates were unblinded and used to design and define the targets on the final panel. Percent agreement from this unblinded Development Set was 99.1% (215/217) (Table S2).

The remaining 320 blinded isolates were subcultured onto solid media and used to evaluate the accuracy of the AFB Primary Panel. The percent agreement for the blinded evaluation set was 96.2% (308/320). Twelve isolates resulted in no identification (3.8%, 12/320), despite repeat testing. The overall sensitivity was 94.3% (199/211) for all organisms represented on the panel, and it ranged from 90.0% to 100% for individual targets (Table 2). Specificity was assessed using negative culture media, non-target mycobacteria, and routine bacteria not represented on the panel. Negative media were tested to confirm clean matrix and no cross-reactivity is observed in any media types. Overall, specificity for identification was 99.1% (108/109), with a range of 94.4–100% for each individual target (Table 2).

**TABLE 2:** Accuracy results for identification using the AFB Primary Panel^*g*^

Target		No. of isolates tested	Reference standard isolates tested		MALDI-TOF mass spectrometry				Percent (%, confidence interval)	
			Positive	Negative	TP	FP	TN	FN	Sensitivity	Specificity
*M. abscessus* subsp. *abscessus*		23	23	297	21	0	297	2	91.3	100.0
									(72.0, 98.7)	(98.5, 100)
*M. abscessus* subsp. *bolletii*		10	10^*a*^	310	10	2^*b*^	308	0	100.0	99.4
									(67.9, 100)	(97.5, 99.9)
*M. abscessus* subsp. *massiliense*		20	20	300	18	0	300	2	90.0	100.0
									(68.7, 98.4)	(98.4, 100)
*M. avium sensu stricto*		20	20	300	18	0	300	2	90.0	100.0
									(68.7, 98.4)	(98.4, 100)
*M. intracellulare* subsp. *chimaera*		20	20	300	20	0	300	0	100.0	100.0
									(81.2, 100)	(98.4, 100)
*M. avium* complex, other		35	35^*c*^	285	34	0	285	1	97.1	100.0
									(84.2, 99.9)	(98.4, 100)
*M. chelonae*		24	24	296	23	1^*d*^	295	1	95.8	99.7
									(78.1, 99.9)	(78.1, 99.9)
*M. kansasii*		20	20	300	19	0	300	1	95.0	100.0
									(74.5, 99.9)	(98.4, 100)
*M. tuberculosis* complex		39	39^*e*^	281	36	0	281	3	92.3	100.0
									(78.9, 98.1)	(98.3, 100)
Negatives	Other Mycobacteria	51	0	51	0	0	51	0	N/A	100.0
										(91.6, 100)
	Other bacteria	18	0	18	0	1^*f*^	17	0	N/A	94.4
										(72.4, 99.9)
	Negative solid media (7H11)	40	0	40	0	0	40	0	N/A	99.1
										(89.6, 100)

^*a*^ All were whole-genome sequenced.

^*b*^ Identified as *M. abscessus* subsp. *abscessus.*

^*c*^ 30 *M*. *intracellulare* subsp. *intracellulare*, 3 *M*. *bouchedurhonense,* and 2 *M*. *colombiense* as determined by *rpoB* and/or 16S gene sequencing. The *M. bouchedurhonense* isolate was identified as “*M. intracellulare*” by the LPA; because of known issues with detection of this species, *rpoB* identification was used for final identification.

^*d*^ Resulted as No Identification.

^*e*^ Of these, 20/39 were *M. tuberculosis*, 9/39 were *M. bovis* BCG, 1/39 were *M. africanum*, 7/39 *M*. *bovis*, and 2/39 *M*. *tuberculosis* complex that were unable to be differentiated by the GenoType MTBC VER 1.X.

^*f*^ Identified as *Nocardia veterana.*

^*g*^ TP: True Positive, FP: False Positive, TN: True Negative, FN: False Negative, N/A: Not applicable.

Sensitivity for *erm* (41), *rrl,* and *rrs,* respectively, was 100% (24/24), 91.3% (21/23), and 100% (15/15) and specificity was 100% (6/6), 100% (116/116), and 99.2% (123/124), respectively, compared to reference standard methods (Table 3). False-positive and false-negative gold standard drug resistance results were confirmed by phenotypic antibiotic susceptibility testing.

**TABLE 3:** Accuracy for drug resistance targets using the AFB Primary Panel^*c*^

Target	Originating organism	No. of isolates tested	Reference standard isolates tested		MALDI-TOF mass spectrometry				Percent (confidence interval)	
			Positive	Negative	TP	FP	TN	FN	Sensitivity	Specificity
*erm* (41)	*M. abscessus* subsp. *abscessus*	20	24	6	24	0	6	0	100 (87.9, 100)	100 (55.7, 100)
	*M. abscessus* subsp. *bolletii*	10								
*rrl*	*M. abscessus* subsp. *abscessus*	23	23	116	21	0	116	2^*a*^	91 (72.0, 98.7)	100 (93.5, 99.1)
	*M. abscessus* subsp. *bolletii*	10								
	*M. abscessus* subsp. *massiliense*	20								
	*M. avium sensu stricto*	20								
	*M. avium* complex, other	30								
	*M. chelonae*	20								
	*M. intracellulare* subsp. *chimaera*	20								
*rrs*	*M. abscessus* subsp. *abscessus*	23	15	124	15	1^*b*^	123	0	100 (76.1, 100)	99 (95.1, 99.9)
	*M. abscessus* subsp. *bolletii*	10								
	*M. abscessus* subsp. *massiliense*	20								
	*M. avium sensu stricto*	20								
	*M. avium* complex, other	20								
	*M. chelonae*	20								
	*M. intracellulare* subsp. *chimaera*	20								

^*a*^ 1 isolate was *M. intracellulare* subsp. *intracellulare*, and 1 was from *M. avium*. Both were reported as “Not defined.”

^*b*^ 1 *M. avium* did not have a mutation by line probe assay but a mutation was detected by PCR/MALDI-TOF MS in position 1408.

^*c*^ TP: True Positive, FP: False Positive, TN: True Negative, FN: False Negative.

To assess cross-reactivity, we evaluated a wide range of isolates not represented on the panel (*n* = 85). Of these, one isolate was cross-reactive: *Nocardia veterana* was identified by *rpoB* sequencing and was identified as *M. avium* complex by the AFB Primary Panel (Table S1).

False positives detected during the evaluation of the identification targets were confirmed by testing the same isolate's lysate with the reference standard method of identification for the organism.

Limit of detection testing of identification targets showed that the lowest limit of detection was for *M. abscessus* subsp. *massiliense* (2.2 × 10^3^ CFU/mL) while *M. abscessus* subsp. *abscessus* had the highest limit of detection (1.5 × 10^7^ CFU/mL). For drug resistance targets, the limit of detection for *erm* (41), *rrl,* and *rrs* was 9.9 × 10^6^, 4.6 × 10^6^, and 4.4 × 10^6^ CFU/mL, respectively (Table 4).

**TABLE 4:** Limit of detection of targets represented on the AFB Primary Panel

Target	LOD (CFU/mL)^*b*^	Percent (%) detected
*M. avium sensu stricto*	8.0 × 10^5^	100
*M. avium* complex, other	1.6 × 10^6^	100
*M. intracellulare* subsp. *chimaera*	6.5 × 10^6^	100
*M. abscessus* subsp. *abscessus^a^*	1.5 × 10^7^	97
*M. abscessus* subsp. *bolletii*	5.2 × 10^4^	100
*M. absessus* subsp. *massiliense*	2.2 × 10^3^	100
*M. chelonae*	5.0 × 10^5^	100
*M. kansasii*	1.8 × 10^5^	100
*M. tuberculosis* complex	1.3 × 10^4^	100
*rrl*	4.6 × 10^6^	98
*rrs*	4.9 × 10^6^	98
*erm* (41)	9.9 × 10^6^	96

^*a*^ Average of two isolates tested, one showing susceptibility and one showing resistance in all three drug resistance markers.

^*b*^ CFU/mL: colony forming units per milliliter.

## DISCUSSION

Here, we demonstrate the robust performance of a novel PCR/MALDI-TOF MS platform for the detection and identification of NTM and associated drug resistance markers. The AFB Primary Panel was designed to detect eight common NTM, *M. tuberculosis* complex, and three markers of drug resistance and was shown to be highly accurate (≥96% agreement) compared to reference standard methods.

Assays for complete species/subspecies identification of common NTM are not readily available in the United States (U.S.). For instance, hybridization probes (i.e., AccuProbes, Hologic, Marlborough, MA, USA) were commonly used for mycobacterial identification (22–24) but were discontinued in December 2022 (25). Despite their widespread use, hybridization probes could only identify five NTM targets and did not have the resolution to detect most species within the *M. avium* complex or *M. abscessus* subspecies.

Similarly, other molecular methods for identification of NTM isolates currently include real-time PCR, line probe assays (12, 23, 26–28), and sequencing of conserved genes (22, 24, 27, 29–31). However, none of these assays are FDA-approved or cleared, making it challenging for laboratories in the U.S. to acquire, validate, and perform molecular assays for NTM identification (32).

Multiplex real-time PCR is often only successful for a small number of targets per reaction well because the fluorescent detection dyes are limited and/or overlap with each other (33). Therefore, additional methods can be applied to improve multiplexing capabilities, such as nested PCR, or detection using hybridization on a membrane (such as line probe assays) (34). Nested PCR assays detecting more than five targets typically use complicated microfluidics in single use cartridges (34–36). These assays are expensive, have relatively low throughput, and not easily available for NTM. Line probe assays are PCR-based hybridization assays that can identify drug resistance markers as well as multiple mycobacteria to the species and subspecies level. These may be validated in the U.S. as laboratory-developed tests but are labor-intensive, time consuming, and subjective.

Sequencing assays are the gold standard method of identification for mycobacteria. Typically, Sanger sequencing is used on single genes, such as 16S, *hsp*65, ITS, or *rpoB*, but these assays require significant technical expertise and equipment and are not routinely performed in laboratories (22, 31, 37, 38).

Another method currently used for mycobacterial identification is MALDI-TOF MS (8, 27, 39–41). This platform is routinely used on cultured organisms and detects ribosomal protein fingerprints (8, 39, 41–45). This technique is extremely useful in microbiology laboratories because of its breadth of identification (including mycobacterial species), rapid turn-around time, low operational cost, and short hands-on time. On the other hand, MALDI-TOF MS from isolates and cultures cannot currently identify mycobacterial subspecies, differentiate some closely related organisms within complexes, or detect drug resistance markers (8, 22). These limitations can be clinically important. For instance, *M. abscessus* subsp. *massiliense* is typically susceptible to clarithromycin due to the absence of a functional *erm* (41) gene, while *M. abscessus* subsp. *bolletii* is typically resistant to clarithromycin due to the presence of *erm* (41). Similarly, full identification of *M. intracellulare* subsp. *chimaera* can be helpful in assessing potential nosocomial infection.

Instead of using MALDI-TOF MS directly on cultured organisms, the AFB Primary Panel assay described here uses MALDI-TOF MS to detect PCR amplicons. By combining technologies, the AFB Primary Panel allows for differentiation of closely related species and subspecies and considerable multiplexing (12 targets) as well as detection of both drug resistance and identification targets within a single reaction. It can identify single nucleotide polymorphisms (SNPs) since detection probe extensions by different ddNTPs will have different molecular weights. As an example, substitution of A to G in position 1408 of 16S rRNA gene sequence is detectable and corresponds to aminoglycoside resistance (12, 46, 47). Furthermore, PCR/MALDI-TOF MS is a high-throughput assay and can be performed in a 96-well or 384-well format. It also does not require manual test interpretation and the cost per test depends on inexpensive reagents (no costly fluorophores or tags).

On the other hand, there are several limitations to this assay and platform. For instance, the limits of detection for the targets on the assay were relatively high (between 10^4^ and 10^7^ CFU/mL), suggesting that testing may be limited to pure isolates instead of direct testing from sputum samples, especially smear negative samples. Similarly, the relatively low sensitivity for MTBC suggests that the assay is not suitable as a primary screening tool for MTBC but may aid in identifying unanticipated isolates. In addition, designing highly specific molecular probes for closely related organisms can prove difficult. For example, *Nocardia veterana* was identified as *M. avium* complex, which could potentially be to sequence similarities or potential gene transfer events. This discrepant result could also be due to a co-infection, which is not identifiable by this assay. The 12 isolates not identified in the challenge set may have been due to the high limit of detection, poor DNA lysis, or from genetic differences within the target regions being evaluated.

In addition to technical limitations, this methodology has shown to be difficult to implement into the clinical laboratory, largely due to the technical learning curve, the challenge of an open system, and complexity of the multi-step PCR and the MassArray instrument. Furthermore, the time from nucleic acid extraction to results can be ~4–10 hours or more, which could potentially result in a -day turn-around time.

*M. intracellulare* subsp. *intracellulare* is not a distinct target on this panel. Importantly, the line probe comparator does contain this target; however, this is not accurate, for it will incorrectly include isolates such as *M. intracelllulare* subsp. *yongonense* (which is assigned by some experts as a different subspecies) (17, 18)*, M. bouchedurhonense*, *M. marseillense, M. arosiense,* and *M. timonense* as “*M. intracellulare*” (20, 21). To avoid missing these organisms, the AFB Primary Panel instead uses the term “*M. avium* complex, other” so that the laboratory may utilize additional methods for full identification, if necessary.

PCR/MALDI-TOF MS methods have been previously assessed for other respiratory pathogens (33), including potential differentiation of *M. tuberculosis* complex (48). However, to our knowledge, this is the first PCR/MALDI-TOF panel that targets common NTM and relevant markers of drug resistance. Here, we show that it can be a useful tool for full identification of common mycobacteria.

## SUPPLEMENTAL MATERIAL

Supplementary material spectrum.01638-24-s0001.pdf Tables S1 and S2.

ASM does not own the copyrights to Supplemental Material that may be linked to, or accessed through, an article. The authors have granted ASM a non-exclusive, world-wide license to publish the Supplemental Material files. Please contact the corresponding author directly for reuse.
