# Supplementary material for: MALDI-TOF mass spectrometry from nucleic acid: development and evaluation of a novel platform for identification of mycobacteria and detection of genetic markers of resistance
Source: Microbiol Spectr. 2024 Aug 27;12(10):e01638-24. doi: 10.1128/spectrum.01638-24 (PMC11448058; doi:10.1128/spectrum.01638-24)
Supplement: Supplementary material — Tables S1 and S2. [file spectrum.01638-24-s0001.pdf]

Supplementary Table 1. Cross reactivity with targets identified by the AFB Primary Panel

Organism	Number Tested	Number cross reactive
Corynebacterium sp.[a]	3	
E. coli	2	
Gordonia sp.[a]	1	
Gordonia sputi	2	
M. arosiense	1	
M. brisbanense	1	
M. conceptionense	1	
M. farcinogenes	1	
M. fortuitum group	20	
M. franklinii	4	
M. goodii	2	
M. gordonae	2	
M. haemophilum	2	
M. immunogenum	6	
M. lentiflavum	1	
M. mageritense	2	
M. marinum	2	
M. marseillense	3	
M. mucogenicum	2	
M. neoaurum	1	
M. neworleansense	1	
M. phocaicum	2	
M. porcinum	1	
M. sengalense	2	
M. septicum	1	
M. setense	1	
M. simiae	1	
M. smegmatis	1	
M. szulgai	1	
M. timonense	2	
M. vulneris	1	
M. wolinskyi	1	
M. xenopi	1	
Nocardia sp.[a]	6	
Nocardia veterana	1	1
S. aureus	1	
Tsukamurella tyrosinosolvens	1	
Tsukamurella pulmonis	1	
Total	85	1

[a]No further speciation was performed

Supplementary Table 2. Accuracy of Identification Results using the Development Set

Target	Reference Standard Samples Tested		MALDI-TOF Mass Spectrometry				Percent (Confidence Interval)	
	Positive	Negative	TP	FP	TN	FN	Sensitivity	Specificity
M. abscessus subsp. *abscessus*	42	175	42	2	175	0	100.0 (90.0, 100)	98.9 (95.7, 99.9)
M. abscessus subsp. *bolletii*	9	208	9	1	208	0	100.0 (65.5, 100)	98.5 (97.1, 99.9)
M. abscessus subsp. *massiliense*	24	193	24	0	193	0	100.0 (83.69, 100)	100.0 (83.7, 100)
M. avium sensu stricto	21	196	20	1	196	1	90.0 (75.6, 99.9)	98.0 (75.6, 99.9)
M. intracellulare subsp. *chimaera*	17	200	17	1	200	0	100.0 (78.4, 100)	98.1 (78.4, 100)
M. avium complex, other	32	185	32	0	185	0	100.0 (87.3, 100)	100.0 (87.3, 100)
M. chelonae	17	200	16	0	200	1	100.0 (71.0, 99.9)	100.0 (71.1, 99.9)
M. kansasii	15	202	15	0	202	0	100.0 (76.1, 100)	98.1 (76.1, 100)
M. tuberculosis complex[a]	20	197	20	0	197	0	100.0 (38.3, 100)	100.0 (97.7, 100)
Other Mycobacteria[b]	20	197	20	1	197	0	N/A	100 (96.9, 99.9)

TP: True Positive, FP: False Positive, TN: True Negative, FN: False Negative, N/A: Not applicable

[a]1 *M. bovis*, 1 *M. bovis* BCG, 17 *M. tuberculosis*, 1 *M. tuberculosis* complex not further differentiated.

[b]19 *M. fortuitum* and 1 *M. fortuitum* group not further differentiated.
